# Candidate Gene Analysis of Femoral Neck Trabecular and Cortical Volumetric Bone Mineral Density in Older Men

**DOI:** 10.1359/jbmr.090729

**Published:** 2009-07-13

**Authors:** Laura M Yerges, Lambertus Klei, Jane A Cauley, Kathryn Roeder, Candace M Kammerer, Kristine E Ensrud, Cara S Nestlerode, Cora Lewis, Thomas F Lang, Elizabeth Barrett-Connor, Susan P Moffett, Andrew R Hoffman, Robert E Ferrell, Eric S Orwoll, Joseph M Zmuda

**Affiliations:** 1 Epidemiology, University of Pittsburgh Pittsburgh, PA, USA; 2 Psychiatry, University of Pittsburgh Pittsburgh, PA, USA; 3 Statistics, Carnegie Mellon University Pittsburgh, PA, USA; 4 Genetics, University of Pittsburgh Pittsburgh, PA, USA; 5 Medicine, University of Minnesota Minneapolis, MN, USA; 6 University of Alabama at Birmingham Birmingham, AL, USA; 7 Radiology, University of California–San Francisco San Francisco, CA, USA; 8 Department of Family and Preventive Medicine, University of California–San Diego La Jolla, CA, USA; 9 Veterans Affairs, Palo Alto Health Care System and Stanford University Palo Alto, CA, USA; 10 Medicine, Oregon Health & Science University Portland, OR, USA

**Keywords:** osteoporosis, Genetics, BMD, men, qCT

## Abstract

In contrast to conventional dual-energy X-ray absorptiometry, quantitative computed tomography separately measures trabecular and cortical volumetric bone mineral density (vBMD). Little is known about the genetic variants associated with trabecular and cortical vBMD in humans, although both may be important for determining bone strength and osteoporotic risk. In the current analysis, we tested the hypothesis that there are genetic variants associated with trabecular and cortical vBMD at the femoral neck by genotyping 4608 tagging and potentially functional single-nucleotide polymorphisms (SNPs) in 383 bone metabolism candidate genes in 822 Caucasian men aged 65 years or older from the Osteoporotic Fractures in Men Study (MrOS). Promising SNP associations then were tested for replication in an additional 1155 men from the same study. We identified SNPs in five genes (*IFNAR2*, *NFATC1*, *SMAD1*, *HOXA*, and *KLF10*) that were robustly associated with cortical vBMD and SNPs in nine genes (*APC*, *ATF2*, *BMP3*, *BMP7*, *FGF18*, *FLT1*, *TGFB3*, *THRB*, and *RUNX1*) that were robustly associated with trabecular vBMD. There was no overlap between genes associated with cortical vBMD and trabecular vBMD. These findings identify novel genetic variants for cortical and trabecular vBMD and raise the possibility that some genetic loci may be unique for each bone compartment. © 2010 American Society for Bone and Mineral Research

## Introduction

Bone mineral density (BMD) is an established determinant of bone strength and osteoporotic fracture risk. BMD assessed by conventional dual-energy X-ray absorptiometry (DXA) measures the total bone mineral content in a projected area (integral areal BMD) and cannot directly measure other skeletal features that also may contribute to bone strength, such as the relative amounts of cortical and trabecular bone. In contrast, quantitative computed tomography (QCT) provides a direct measure of cortical and trabecular volumetric BMD, both of which may contribute to bone strength and fracture risk.(1,2)

BMD is a highly heritable trait that is under context-specific genetic regulation.(3) For example, the individual genetic factors contributing to BMD variation may differ between women and men, between skeletal sites, and between trabecular and cortical bone.(4–11) Volumetric BMD (vBMD) for both compartments is heritable, with heritability estimates of 17% to 42% for cortical vBMD and 59% to 73% for trabecular vBMD.(9–11) However, the vast majority of studies in humans have searched for genetic variants associated with DXA measures of integral BMD, and most have focused on women. To better understand the genetic determinants of compartment-specific vBMD in humans, we explored the association of common genetic variation in 383 candidate genes with cortical and trabecular vBMD of the femoral neck in 1977 men from the Osteoporotic Fractures in Men Study (MrOS).

## Methods

### Genotyping sample

This study used a two-stage genotyping design with two independent samples of older Caucasian men from the MrOS. The parent study has been described, but in brief, MrOS is a prospective cohort study of skeletal health in 5995 community-dwelling men age 65 and older.(12,13) Men were recruited at six clinical centers across the United States. At the time of entry into the study, the men were ambulatory and had at least one native hip.

The genotyping samples were comprised of Caucasian men who had vBMD measurements at the femoral neck and did not report taking bone-altering medications (e.g., androgens, antiandrogens, or oral corticosteroids) or osteoporosis treatment. We used an internal replication study design by dividing our sample in two parts and conducting our genotyping in two phases. The first phase of genotyping was completed on a discovery sample that included 822 men who were selected without regard to their BMD level from the Minneapolis and Pittsburgh clinic sites. Promising single-nucleotide polymorphism (SNP) associations identified in the discovery sample then were tested for replication in a validation sample consisting of 1156 additional men from the four other MrOS clinic sites (Birmingham, Palo Alto, Portland, and San Diego).

### Participant characteristics

Clinic staff documented participant characteristics, including age and medical history. Participants were asked to bring all medications used in the past 30 days to their clinic visit, where clinic staff recorded medication use information in an electronic database (UCSF Coordinating Center, San Francisco, CA, USA). Height was measured by Harpenden stadiometer, and weight was measured by balance-beam scale or digital scale with participants wearing light clothing and no shoes.

### Volumetric BMD

vBMD of the femoral neck was measured using QCT. The first 65% of the MrOS cohort (and all ethnic minorities, regardless of when they entered the study) were referred for QCT scans. Men having had a hip replacement were not eligible for a hip scan. Those who received a QCT scan were similar to those who did not.(14)

To measure vBMD at the femoral neck, a QCT scan of the pelvic region (from the femoral head to 3.5 cm below the lesser trochanter) was acquired at settings of 80 kVp, 280 mA, 3-mm slice thickness, and 512 × 512 matrices. Images were acquired using a GE Prospeed (Birmingham), GE Hispeed Advantage (Minneapolis), Philips MX-8000 (Palo Alto), Siemens Somatom +4 (Pittsburgh), Philips CT-Twin (Portland), Toshiba Acquilion (Portland) site, or Picker PQ-5000 scanner (San Diego). Differences between the clinic sites exist, and clinic site is included as a covariate in all models.

Each participant scan included a calibration standard of three hydroxyapatite concentrations (150, 75, and 0 mg/cm^3^). Images were converted from the native scanner Hounsfield units (HU) to equivalent concentration (g/cm^3^) of calcium hydroxyapatite contained in the calibration standard. Regions of interest (ROI) in the left proximal femur were identified in QCT images re-formatted along the neutral axis of the femoral neck. The periosteal boundary of the femur was determined with a threshold-based region-growing algorithm. Using this boundary, the cross-sectional area in each slice along the neutral axis of the femoral neck between the proximal femoral neck and the lateral edge of the trochanter was calculated, and the minimum and maximum areas were determined. The femoral neck ROI was defined as the portion of the neck extending from the slice with minimum cross-sectional area (medial boundary) to a point 25% of the distance toward the maximal cross-sectional area. Integral volume of the ROI was computed as the total volume within the periosteal boundary. A trabecular volume of the ROI was obtained by applying an erosion process to the integral volume to retain the same shape in a region fully contained within the medullary space. The cortical volume then was defined by applying a threshold of 0.35 g/cm^3^ to all voxels between the periosteal boundary and the outer boundary of the trabecular volume. All QCT scans were transferred to the University of California–San Francisco for processing and central review. Volumetric BMD for trabecular and cortical compartments was computed over all voxels in the respective volumes. In a group of postmenopausal women, coefficients of variation ranged from 0.6% to 3% for vBMD measures.(15)

### Candidate gene and SNP selection

Candidate genes for vBMD were identified using evidence from several sources, including literature searches (PubMed, http://www.ncbi.nlm.nih.gov/sites/entrez?db=PubMed), evidence of gene expression in a normal human trabecular bone cells (Skeletal Gene Database, sgd.nia.nih.gov [no longer available], and NCBI UniGene, http://www.ncbi.nlm.nih.gov/sites/entrez?db=unigene), genes with functions of interest such as “regulation of bone mineralization” or “skeletal development” (Entrez Gene, http://www.ncbi.nlm.nih.gov/sites/entrez?db=gene, and Amigo, amigo.geneontology.org/cgi-bin/amigo/go.cgi), genes with a skeletal phenotype in mice (Jackson Laboratory Mouse Genome, http://www.informatics.jax.org), and genes implicated in human skeletal disorders (Online Mendelian Inheritance in Man, http://www.ncbi.nlm.nih.gov/omim). From these resources, 383 candidate genes were identified for genotyping.

For the first phase of genotyping (discovery sample), publically available data were interrogated for SNP variation in the region surrounding the candidate gene. This was accomplished by first creating a reference SNP panel of variants with a minor allele frequency (MAF) of at least 5% in the genomic region spanning 30 kb upstream of the transcription start site and 10 kb downstream of the candidate gene transcript. SNPs were selected using phase I of the International HapMap Project (http://www.hapmap.org), which was the current version at the time of discovery SNP selection.(16) Tag SNPs were selected from this reference SNP panel using a pairwise correlation method (*r*^2^ ≥ 0.80).(17) Candidate genes located near each other on the chromosome were tagged as a unit spanning all loci of interest (such as *IGFBP2* and *IGFBP5*, which are located only 7.6 kb from each other on chromosome 2). In addition to tag SNPs, potentially functional SNPs (nonsynonymous coding variants, SNPs predicted to alter a transcription factor binding site and putative exon splice enhancers) with MAF ≥ 1% were selected for genotyping. These putative functional SNPs were selected using either PupaSNP (pupasuite.bioinfo.cipf.es/) or Promolign (polly.wustl.edu/promolign/main.html).(18,19)

In the second phase of the project (the validation sample), promising SNP associations from the discovery sample were replicated. We arbitrarily defined our candidate genes as potentially interesting for follow-up if they contained at least one SNP with a *p* value of .015 or less in the discovery sample. We identified 72 candidate genes for cortical vBMD and 75 for trabecular vBMD and genotyped any SNP with a *p* value of .05 or less within those genes in the validation sample. This two-tiered strategy for selecting SNPs for follow-up was done to more comprehensively interrogate promising candidate genes in the validation sample than only replicating findings with a discovery *p* value of .015 or less.

### Genotyping

Genomic DNA was extracted from frozen whole blood specimens using the Flexigene protocol (Qiagen, Valencia, CA, USA). Genotyping in the discovery sample and most of the genotyping in the validation sample were completed using the Illumina Golden Gate custom assay. Blind duplicate samples and internal controls were included to ensure reproducibility. For the discovery sample, we observed 100% reproducibility among the four internal controls run on each plate and 99.9% reproducibility among the 37 duplicate participant samples. In the validation sample, we observed 99.9% reproducibility among the four internal controls run on each plate and 99.9% reproducibility among the 26 blind duplicate samples. To ensure maximum genotyping completeness in the validation sample, loci of interest that could not be genotyped successfully using the Illumina Golden Gate assay were genotyped using one of two platforms: the TaqMan allelic discrimination assay system (Applied Biosystems, Foster City, CA, USA) on a 7900HT Real-time PCR instrument with probes and reagents purchased from Applied Biosystems or the Sequenom MassARRAY iPLEX Gold technology instrument (Sequenom, Inc., San Diego, CA, USA) with PCR primers purchased from Invitrogen (Carlsbad, CA, USA). Participant samples were run in duplicate for these platforms, and an average reproducibility of 99.8% and 99.9% was observed for TaqMan and Sequenom instruments, respectively.

Several participants' samples were excluded from these analyses because they had a low genotyping call rate (excluded if less than 85% of SNPs called per participant, *N* = 14) or were highly correlated with another sample indicating relatedness (*N* = 13). Related individuals were identified by looking at pairwise identity-by-state (IBS) distances and identifying pairs with higher than expected IBS. Relatedness of these outlier pairs was confirmed with clinic staff. Before analysis of the discovery sample, 500 loci were dropped based on predefined quality control parameters. Specifically, loci in the discovery sample that had an observed minor allele frequency of less than 1% (*N* = 129), that did not conform to the expectations of Hardy-Weinberg equilibrium (*P* < .005, *N* = 123), or that had a low call rate (>85% of samples missing per SNP, *N* = 248) were excluded from statistical analysis.

We genotyped, on average, 1 SNP per 13 kilobase pairs (kbp) across each candidate gene region (range: 1 SNP/3 kbp – 1 SNP/97 kbp). The 4108 of 4608 SNPs genotyped successfully tagged, on average, 64% of the SNPs with an MAF > 5% in phase II of HapMap (range per gene: 1% to 100%). Of the HapMap reference SNPs captured by our tag SNP set, the average correlation with the selected tag SNP was 0.97.

### Statistical analysis

Uncorrelated SNPs (*r*^2^ < 0.04) were selected by using a pairwise correlation method for the discovery, validation, and pooled samples, and these uncorrelated SNPs were used in subsequent analysis of population stratification.(17) Population stratification was assessed initially using Structure.(20) There was little evidence of population stratification, but we accounted for potential fine-scale population stratification in subsequent analyses using a principal-components method.(21) The first principal component explained less than 0.5% variation for cortical or trabecular vBMD in either the discovery or validation sample.

Genetic analyses assumed an additive and recessive model. Linear regression was used to test for an additive association between the number of copies of the minor allele and vBMD. Regression methods were implemented for the recessive model to test if individuals with two copies of the minor allele differed from those with the other genotypes. For instance, where a SNP had fewer than 10 individuals with the rare genotype, only the additive model was tested to minimize spurious findings based on small genotype-specific sample sizes. Analyses were adjusted for patient age, clinic site, and the first principal component from the population-stratification analysis. SNPs significantly associated in both the discovery and validation samples (*p* < .05) and having an association in the same direction for both genotyping samples (a positive or negative regression coefficient for both samples), regardless of the genetic model, were considered replicated findings. Replicated SNP associations were examined further in the pooled sample of 1977 individuals from the discovery and validation samples. Linear regression was used in the pooled sample to test for both additive and recessive models. The pooled sample was adjusted for participant age, clinic site, and the first principal component from the population-stratification analysis. Additional adjustment for height and weight was conducted in the pooled sample to determine if body size attenuated the relationship between genotype and vBMD. Linear regression analysis was used to determine the amount of phenotypic variation explained by the significant replicated SNPs. Correlation between individual SNPs in the model (*r*^2^) was assessed to minimize collinearity in the model. For instance, where SNPs in the model were highly correlated, the SNP with the most missing genotypes was dropped from the regression modeling.

## Results

The average age of the 822 men in the discovery sample was 73 years (range 65 to 100 years) and did not differ from the validation sample (Table 1). Participants in the validation sample were slightly taller, weighed less, had a lower body mass index (BMI), and had lower cortical and trabecular vBMD at the femoral neck (*p* < .001 for all).

**Table 1:** Characteristics of Older Caucasian Men in the Genotyping Samples

	Discovery sample N = 821	Validation sample N = 1156	Pooled sample N = 1977
Age (years)	73 (5.7)	74 (6.0)	74 (5.9)
Height (cm)	173.6 (6.8)	174.9 (6.7)*	174.3 (6.7)
Weight (kg)	85.3 (14.1)	82.9 (12.5)*	83.9 (13.2)
BMI (kg/m^2^)	28.3 (4.1)	27.1 (3.6)*	27.6 (3.8)
Femoral neck cortical volumetric BMD (g/cm^3^)	0.532 (0.055)	0.520 (0.065)*	0.525 (0.061)
Femoral neck trabecular volumetric BMD (g/cm^3^)	0.086 (0.044)	0.062 (0.040)*	0.072 (0.043)

* *p* < .001.

**Fig. 1:**
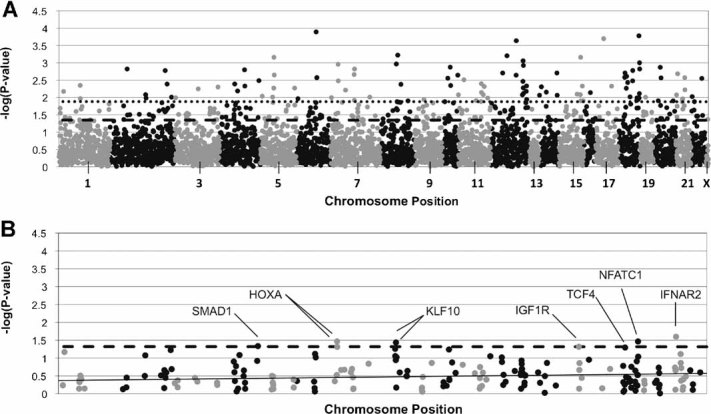
SNP association results for femoral neck cortical volumetric bone mineral density. Association results for cortical volumetric bone mineral density are presented for the first phase of genotyping: (*A*) discovery sample; (*B*) validation sample. Specifically, the –log of the *p* value observed is presented on the *y* axis. The most significant result of the two models tested (either additive or recessive) is presented for each SNP. The SNPs are ordered across the *x* axis by chromosome and the basepair position on the chromosome. Odd-numbered chromosomes and the X chromosome are presented in gray. Even-numbered chromosomes are presented in black. In (*A*), the dark dashed line represents *p* = .015 and the dotted line represents *p* = .05. In (*B*), the dotted line represents *p* = .05, and SNPs with *p* values of .05 or less are labeled with the gene symbol that they lie in.

Of the 4108 SNPs genotyped in the discovery analysis, 191 SNPs in 72 genes were associated with cortical vBMD and were genotyped in the validation sample (Fig. 1*A*). Of these 191 SNPs, 7 SNPs in 5 genes were consistently associated with cortical vBMD in the validation sample (see Fig. 1*B*). One SNP was identified in the *IFNAR2* (rs2834160), *NFATC1* (rs177820), and *SMAD1* (rs1874572) gene regions, whereas two SNPs were identified in the *HOXA* gene cluster (rs6951180 and rs6964896) and *KLF10* gene region (rs3133287 and rs1434278). In addition, one SNP in *IGF1R* (rs3784606) and one SNP in *TCF4* (rs7240986) were significant in both the discovery and validation samples, but the direction of the association was inconsistent.

A pooled analysis was conducted to determine the amount of variation in cortical vBMD that each of the seven significant SNPs explained (Table 2). The most significant SNP association was with rs177820 in the *NFATC1* gene region (*p* = 4 × 10^−4^) that explained 0.5% of the phenotypic variation in cortical vBMD. For this SNP, men with the less common *GG* genotype had 1.2% lower cortical vBMD than those with the *AA* genotype. Additional adjustment for height and weight did not attenuate the relationship.

**Table 2:** Significant SNP Associations With Cortical Volumetric BMD at the Femoral Neck

				Discovery sample		Validation sample		Pooled sample				
						
				Adjustment 1^b^		Adjustment 1^b^		Adjustment 1^b^		Adjustment 2^c^		
							
Gene	SNP	Allele	Frequency^a^	*β*	*P* value	*β*	*P* value	*β*	*P* value	*β*	*P* value	*r*^2^ d
*HOXA*^e^	rs6951180	A→G	0.13	0.013	.001 ^add^	0.027	.034 ^rec^	0.025	.017 ^rec^	0.023	.026 ^rec^	0.002
*HOXA*^e^	rs6964896	C→A	0.13	0.012	.003 ^add^	0.028	.048 ^rec^	0.025	.025 ^rec^	0.023	.036 ^rec^	0.002
*IFNAR2*	rs2834160	A→G	0.17	0.011	.002 ^add^	0.022	.025 ^rec^	0.024	.001 ^rec^	0.024	.001 ^rec^	0.005
*NFATC1*	rs177820	A→G	0.36	−0.009	.001 ^add^	−0.010	.034 ^rec^	−0.013	4 × 10^−4^ ^rec^	−0.012	.001 ^rec^	0.005
*SMAD1*	rs1874572	A→C	0.33	0.017	.003 ^rec^	0.005	.044 ^add^	0.011	.005 ^rec^	0.011	.006 ^rec^	0.004
*KLF10*	rs1434278	T→A	0.23	0.023	.015 ^rec^	0.006	.037 ^add^	0.006	.002 ^add^	0.006	.001 ^add^	0.005
*KLF10*	rs3133287	G→C	0.17	0.038	.001 ^rec^	0.006	.055 ^add^	0.007	.001 ^add^	0.007	.001 ^add^	0.005

*Note:* Additive (add) and recessive (rec) models were tested for each SNP, and the regression parameter and *p* value from the most significant genetic model (additive or recessive) are shown.

add = *p* value from the additive model; rec = *p* value from recessive model.

a Minor allele frequency in the pooled sample.

b Adjustment 1: Age, clinic site, population substructure.

c Adjustment 2: Age, clinic site, population substructure, height, body weight.

d Amount of variation explained after adjusting for age, site, population substructure, height, body weight.

e *HOXA* genes were tagged as a cluster. They include *HOXA1*, *HOXA2*, *HOXA3*, *HOXA4*, *HOXA5*, *HOXA6*, *HOXA7*, *HOXA9*, *HOXA10*, *HOXA11*, and *HOXA13*.

Individual SNPs explained between 0.2% and 0.5% of the variance in cortical vBMD. Regression models were constructed to determine the amount of variation explained by all significant and replicated SNPs. There was high linkage disequilibrium (LD) between the two SNPs in the *HOXA* gene region (rs6951180 and rs6964896, *r*^2^ = 0.691, *D*' = 0.967) and the two SNPs in *KLF10* (rs3133287 and rs1434278, *r*^2^ = 0.882, *D*' = 1.000); thus rs6964896 and rs1434278 were dropped from the regression modeling to minimize collinearity in the model. The five remaining SNPs in *IFNAR2*, *NFATC1*, *SMAD1*, *HOXA*, and *KLF10* explained 1.8% of the variance (adjusted *r*^2^) in cortical vBMD after accounting for age, clinic, population substructure, height, and weight.

**Fig. 2:**
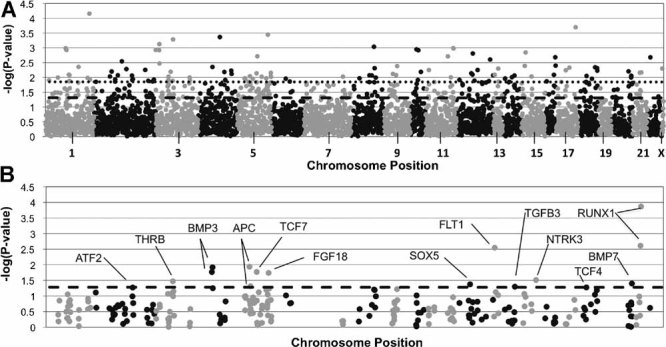
SNP association results for femoral neck trabecular volumetric bone mineral density. Association results for trabecular volumetric bone mineral density are presented for the discovery (*A*) and validation (*B*) samples. Specifically, the –log of the *p* value observed is presented on the *y* axis, and SNPs are ordered across the *x* axis by chromosome and basepair position. The most significant result of the two models tested (either additive or recessive) is presented for each SNP. Odd-numbered chromosomes and the X chromosome are presented in gray. Even-numbered chromosomes are presented in black. In (*A*), the dark dashed line represents *p* = .015, and the dotted line represents *p* = .05. In (*B*), the dotted line represents *p* = .05, and SNPs with *p* values of .05 or less are labeled with the gene symbol that they lie in.

We also identified 255 SNPs in 75 genes that were associated with trabecular vBMD (Fig. 2*A*). We confirmed an association with 12 of these SNPs in nine genes and trabecular vBMD in the validation sample (see Fig. 2*B*). Specifically, associations with one SNP in *ATF2* (rs4972738), *BMP7* (rs6127983), *FGF18* (rs313543), *FLT1* (rs1408245), *TGFB3* (rs7149264), and *THRB* (rs1505289) and two SNPs in *APC* (rs459552 and rs4705573), *BMP3* (rs2903746 and rs6814223), and *RUNX1* (rs2834676 and rs2834694) were replicated in the validation sample (see Fig. 2*B*). In addition, SNPs in *NTRK3* (rs6496469), *SOX5* (rs2133345), *TCF4* (rs618869), and *TCF7* (rs30496) were significantly associated with trabecular vBMD in both the discovery and validation samples, but the direction of association was inconsistent between samples.

Further analysis of the 12 consistently associated SNPs was conducted in the pooled sample. The most significant SNP association for trabecular vBMD was with rs2834694 in *RUNX1* (*p* = 5.3 × 10^−6^) that explained 1.0% of the variance in trabecular vBMD. Individuals with the less common *AA* genotype had a 14% higher trabecular vBMD than men with the *CC* genotype. Statistical adjustment for height and weight in addition to age, clinic, and population substructure did not significantly attenuate the association between the 12 replicated SNPs and trabecular vBMD (Table 3).

**Table 3:** Significant SNP Associations With Trabecular Volumetric BMD at the Femoral Neck

				Discovery sample		Validation sample		Pooled sample				
						
				Adjustment 1^b^		Adjustment 1^b^		Adjustment 1^b^		Adjustment 2^c^		
							
Gene	SNP	Allele	Frequency^a^	*β*	*P* value	*β*	*P* value	*β*	*P* value	*β*	*P* value	*r*^2^ d
*APC*	rs459552	T→A	0.23	0.006	.018 ^add^	0.004	.049 ^add^	0.004	.005 ^add^	0.004	.006 ^add^	0.004
*APC*	rs4705573	A→G	0.48	−0.007	.049 ^rec^	−0.007	.012 ^rec^	−0.007	.002 ^rec^	−0.007	.002 ^rec^	0.005
*ATF2*	rs4972738	G→A	0.37	−0.011	.012 ^rec^	−0.007	.054 ^rec^	−0.009	.001 ^rec^	−0.009	.001 ^rec^	0.005
*BMP3*	rs2903746	A→T	0.18	0.006	.023 ^add^	0.005	.011 ^add^	0.005	.002 ^add^	0.005	.003 ^add^	0.004
*BMP3*	rs6814223	G→A	0.13	0.007	.039 ^add^	0.006	.016 ^add^	0.005	.007 ^add^	0.006	.006 ^add^	0.004
*BMP7*	rs6127983	A→G	0.36	−0.009	.053 ^rec^	−0.007	.040 ^rec^	−0.008	.008 ^rec^	−0.007	.007 ^rec^	0.004
*FGF18*	rs9313543	G→A	0.19	−0.017	.029 ^rec^	−0.005	.018 ^add^	−0.004	.013 ^add^	−0.004	.019 ^add^	0.003
*FLT1*	rs1408245	C→G	0.17	−0.026	.008 ^rec^	−0.006	.003 ^add^	−0.017	.002 ^rec^	−0.016	.003 ^rec^	0.004
*TGFB3*	rs7149264	A→C	0.15	0.006	.034 ^add^	0.004	.050 ^add^	0.005	.008 ^add^	0.005	.011 ^add^	0.003
*THRB*	rs1505289	A→G	0.43	−0.004	.048 ^add^	−0.004	.034 ^add^	−0.004	.003 ^add^	−0.004	.003 ^add^	0.004
*RUNX1*	rs2834676	G→A	0.42	0.005	.030 ^add^	0.010	.003 ^rec^	0.004	.003 ^add^	0.007	.002 ^rec^	0.005
*RUNX1*	rs2834694	C→A	0.49	0.006	.007 ^add^	0.011	1.4 × 10^−4^ ^rec^	0.010	5.3 × 10^−6^ ^rec^	0.010	4.2 × 10^−6^ ^rec^	0.010

*Note:* Additive (add) and recessive (rec) models were tested for each SNP, and the regression parameter and *p* value from the most significant genetic model (additive or recessive) are shown.

add = *p* value from the additive model; rec = *p* value from recessive model.

a Minor allele frequency in the pooled sample.

b Adjustment 1: Age, clinic site, population substructure.

c Adjustment 2: Age, clinic site, population substructure, height, body weight.

d Amount of variation explained after adjusting for age, site, population substructure, height, body weight.

Each of the 12 replicated SNPs individually explained between 0.3% and 1.0% of the variation in trabecular vBMD. None of the 12 replicated SNPs were highly correlated (even those in the same gene region), and all were entered into a regression model. After accounting for age, clinic, population substructure, height, and weight, these 12 SNPs explained 4.0% of the variation in trabecular vBMD.

## Discussion

Studies in humans and animal models suggest that the genetic determinants of trabecular and cortical BMD may differ, but few specific loci have been identified for these bone strength–related traits. To our knowledge, the current study is the first to systematically investigate the association between common genetic variation in candidate genes and vBMD in the trabecular and cortical bone compartments in humans. We identified several genetic variants that were associated robustly with cortical or trabecular vBMD at the femoral neck in a large cohort of older Caucasian men. Specifically, seven SNPs located in five gene regions were consistently associated with cortical vBMD and 12 SNPs in nine genes were consistently associated with trabecular vBMD in two independent samples of men from the same study cohort. No SNP consistently associated with one bone compartment also was associated with the other compartment. These results support the hypothesis that some loci for cortical and trabecular vBMD may be unique for each bone compartment.

To the best of our knowledge, associations of SNPs in *IFNAR2*, *SMAD1*, and *KLF10* with BMD have not been described previously. Evidence specific to cortical bone regulation exists for one of these novel findings, *KLF10*. Krupple-like factor 10 (KLF10) is expressed in osteoblasts and is critical for osteoblast-mediated mineralization and osteoclast differentiation.(22,23) *KLF10* knockout mice have decreased cortical bone thickness and osteocyte number.(24)

We identified a novel association between SNPS in *ATF2* and trabecular vBMD. *ATF2* encodes activating transcription factor 2, a transcription factor that binds to cAMP response elements (CREs) and stimulates CRE-dependent transcription. The *ATF* family plays a role in the expression of skeletal-specific genes and skeletal development,(25) including trabecular bone formation.(26) Indeed, *ATF2* knockout mice lack normal-appearing trabeculae.(27) Animal models also show a trabecular phenotype for two other candidate genes, *BMP3* and *FLT1*, that were associated with trabecular vBMD in this study. BMP3 is one of the most abundant bone morphogenetic proteins (BMPs) in bone and *BMP3* knockout mice have increased trabecular bone.(28,29) *FLT1* encodes the cell-surface receptor for vascular endothelial growth factor that is involved in osteoclastogenesis and osteoblast differentiation.(30–34) *FLT1* null mice have lower trabecular bone volume.(35) We also observed an association between an intronic SNP in the gene encoding thyroid hormone receptor beta (*THRB*) and trabecular vBMD. Adult *THRB* knockout mice have osteoporosis with reduced trabecular and cortical bone, reduced mineralization, and increased osteoclast numbers and activity.(36)

The two variants in the gene encoding adenomatous polyposis coli (*APC*) (rs459552 and rs4705573) were associated with trabecular vBMD at the femoral neck. APC is involved in WNT signaling, is expressed in osteoblasts and osteoclasts, and osteoblast-specific APC deletion increased bone formation in mice.(37–41) The rs459552 variant is of particular interest because it changes asparagine to a valine residue and is located in the ß-catenin downregulation domain of *APC* and may influence WNT signaling.(42,43)

The current findings are consistent with an emerging model whereby some genetic loci for BMD may be specific for cortical and trabecular bone. For example, genome-wide searches in inbred mice have identified loci that appear to be distinctly associated with either cortical or trabecular vBMD(5,8) Furthermore, family studies have found a low genetic correlation between trabecular and cortical vBMD, suggesting different genetic contributions for each bone compartment.(11) Similarly, in the current investigation, none of the replicated genes or SNPs were associated with both cortical and trabecular vBMD at the femoral neck.

We were able to identify more genetic associations and explain a greater fraction of phenotypic variation for trabecular than for cortical vBMD. It is possible that the genetic contribution to trabecular vBMD is greater than for cortical vBMD. Indeed, heritability studies in humans report a lower heritability for cortical vBMD (17% to 42%) than for trabecular vBMD (59% to 73%).(9–11) However, the cortical rim of the femoral neck is thin, and this may have increased measurement error and consequently decreased statistical power at this skeletal region. Future studies of the appendicular skeleton in regions with a larger cortical area (e.g., at the proximal tibia) may help to minimize this issue. Our candidate gene selection also was based on several informatics resources but may have been unknowingly biased toward genes influencing trabecular bone. Future studies of genetic factors for trabecular and cortical vBMD that use hypothesis-free methods (such as genome-wide investigations) may yield further insight on the genetic contributions to each bone compartment.

We were unable to document an association between SNPs in several widely studied candidate genes or genes identified by recent genome-wide association studies (e.g., *COL1A1*, *ESR1*, *LRP5*, *VDR*, *RANKL*, *TNFRSF11B*, and *WNT4*).(44–49) There are several reasons why we may not have been able to replicate these previous associations. First, our study included only men, whereas these past studies have focused primarily on women. Second, most of these prior candidate gene studies measured integral areal BMD by DXA as opposed to cortical and trabecular vBMD by QCT. Third, our study was able to detect an SNP association that explained at least 0.8% of the variation in BMD in the discovery sample with 80% statistical power at alpha = 0.05. Thus we cannot exclude the possibility of a weaker association between SNPs in or near these other genes and vBMD at the femoral neck in older men.

Although the heritability of vBMD is high (estimates range from 40% to 85%), the amount of phenotypic variation in vBMD explained by SNPs in the current study was small (1.7% for cortical vBMD and 4.0% for trabecular vBMD). Although small, the fraction of variation explained by these SNPs individually and collectively is consistent with results from genome-wide association studies of skeletal-related traits such as BMD and height.(48–50) For example, in a recent genome-wide association study of DXA measures of areal BMD, 0.6% and 0.2% of the phenotypic variation in lumbar spine and femoral neck BMD was explained by two SNPs in two genes.(48) Future studies may need to consider other types of polymorphisms such as insertion-deletions, copy-number variants, and less common SNPs (<5% MAF) as well as interactions between genes and between genes and environmental factors in order to better account for the phenotypic variation in vBMD.

Our study has several potential limitations. We focused on older Caucasian men, and our findings may not be generalizable to other populations. Additional genotyping in younger men, ethnically diverse populations, and women will be necessary to further confirm and extend these SNP association findings. Although we selected a relatively large number of genes from bone metabolism pathways in a more comprehensive manner than most candidate gene studies, future GWAS studies of cortical and trabecular vBMD may provide additional insights. Tagging SNP selection was based on phase I of HapMap, which was the current version when the discovery sample genotyping was designed. More recent releases would have provided a denser SNP reference panel from which to select tagging SNPs, and we ultimately would have achieved a superior genotyping coverage of HapMap phase II reference SNPs than the 64% achieved in the current analysis. We also cannot assess the impact that rare variants might have on trabecular and cortical vBMD in our study because we restricted our analysis to tagging SNPs with an MAF ≥5% and potentially functional SNPs with an MAF ≥1%. Finally, it is unclear if the SNPs identified in the current analysis have functional consequences or are merely in linkage disequilibrium with the causal variant(s). Additional studies will be needed to confirm and refine association signals and to identify the causal variants involved.

In conclusion, this is the first large-scale investigation of the potential genetic determinants of cortical and trabecular vBMD in humans at a clinically relevant skeletal site. The two-stage internal replication design identified several novel and reproducible genetic associations. Although additional studies are needed to confirm and extend our findings, the current analysis suggests that the genetic factors contributing to cortical and trabecular vBMD among older men may be, at least to some extent, unique for each bone compartment.
